# Methemoglobin levels in malaria: a systematic review and meta-analysis of its association with *Plasmodium falciparum* and *Plasmodium vivax* infections and disease severity

**DOI:** 10.1038/s41598-024-53741-6

**Published:** 2024-02-08

**Authors:** Manas Kotepui, Aongart Mahittikorn, Polrat Wilairatana, Frederick Ramirez Masangkay, Kinley Wangdi, Kwuntida Uthaisar Kotepui

**Affiliations:** 1 Medical Technology, School of Allied Health Sciences, Walailak University, Thasala, Nakhon Si Thammarat, 80160 Thailand; 2 Department of Protozoology, Faculty of Tropical Medicine, Mahidol University, Bangkok, 10400 Thailand; 3 Department of Clinical Tropical Medicine, Faculty of Tropical Medicine, Mahidol University, Bangkok, 10400 Thailand; 4 Department of Medical Technology, Faculty of Pharmacy, University of Santo Tomas, 1008 Manila, Philippines; 5 QIMR Medical Research Institute, 300 Herston Road, Herston, QLD 4006 Australia

**Keywords:** Oxidized hemoglobin, Methemoglobin, Malaria, *Plasmodium*, Meta-analysis, Malaria, Haematological diseases

## Abstract

Reports indicate that *Plasmodium* infections influence methemoglobin levels**.** However, findings have been inconclusive or have varied across different geographic and demographic contexts. This systematic review and meta-analysis aimed to consolidate existing data regarding the association between *Plasmodium* infections and alterations in methemoglobin levels related to the severity of the infection. A comprehensive literature search of several databases, including Ovid, ProQuest, Embase, Scopus, MEDLINE, and PubMed, was conducted to identify relevant studies that examined methemoglobin levels in patients with malaria. Qualitative synthesis and meta-analysis of the pooled standardized mean difference were conducted to synthesize the differences in methemoglobin levels between: (1) patients with malaria and those without malaria and (2) patients with severe malaria and those with uncomplicated malaria based on various themes including publication year, study design, study area, *Plasmodium* species, age group, symptomatic status, severity status, and method of malaria detection. Of the 1846 studies that were initially identified from the main databases and additional searches on Google Scholar, 10 studies met the eligibility criteria and were selected for this review. The systematic review distinctly highlighted an association between malaria and elevated methemoglobin levels, an observation consistent across diverse geographical regions and various *Plasmodium* species. Furthermore, the meta-analysis confirmed this by demonstrating increased methemoglobin levels in patients with malaria compared to those without malaria (*P* < 0.001, Hedges’ g 2.32, 95% CI 1.36–3.29, *I*^2^ 97.27, 8 studies). Moreover, the meta-analysis found elevated methemoglobin levels in patients with severe malaria compared to those with uncomplicated malaria (*P* < 0.001, Hedges’ g 2.20, 95% CI 0.82–3.58, *I*^2^ 96.20, 5 studies). This systematic review and meta-analysis revealed increased methemoglobin levels in patients with *P. falciparum* and *P. vivax* infections, with a notable association between elevated methemoglobin levels and severe malaria. Future research should focus on elucidating the specific mechanisms by which changes in methemoglobin levels are related to infections by *P. falciparum* and *P. vivax*, particularly in terms of severity, and how these alterations could potentially impact patient management and treatment outcomes.

## Introduction

Malaria, a mosquito-borne infectious disease, remains one of the most prevalent and deadly illnesses worldwide^1^. It is caused by protozoan parasites belonging to the genus *Plasmodium* which are transmitted through the bite of female *Anopheles* mosquitoes^2^. Among the several species of this genus, five are well known to infect humans: *Plasmodium falciparum* (*P. falciparum*), *Plasmodium vivax (P. vivax*), *Plasmodium ovale* (*P. ovale*) (with two distinct species: *P. o. curtisi* and *P. o. wallikeri*), *Plasmodium malariae* (*P. malariae*), and *Plasmodium knowlesi* (*P. knowlesi*)^3,4^. In addition to those main species, *Plasmodium cynomolgi* (*P. cynomolgi*), *Plasmodium inui* (*P. inui*), and other simian malaria parasites can naturally infect humans^5,6^. The spread and prevalence of malaria varies considerably across global regions, with sub-Saharan Africa being disproportionately affected^1^. Malaria can infect individuals of all age groups, including children and adolescents^7,8^, adults^9,10^, and pregnant women^11,12^. Despite concerted efforts to control and eliminate this disease, malaria still results in significant morbidity and mortality, especially in vulnerable populations like children under 5 years^1,13,14^.

Methemoglobin is an oxidized form of hemoglobin containing iron in the ferric [Fe3^+^] form in red blood cells^15^. Under normal physiological conditions, methemoglobin levels are low (< 1%) because of the action of the enzyme cytochrome-b5 reductase^16^. However, methemoglobin levels can rise when these mechanisms are overwhelmed or compromised, resulting in a condition known as methemoglobinemia^15^. Elevated methemoglobin levels can reduce the oxygen-carrying capacity of the blood, leading to a range of clinical symptoms, from cyanosis to more severe respiratory and cardiovascular complications^17,18^.

Studies have indicated a potential association between malaria infection and increased methemoglobin levels^19,20^. In particular, there might be a heightened risk of methemoglobinemia in severe malaria cases, where intravascular hemolysis is common^21^. The increase in methemoglobinemia has been associated with the severity and fatality of malaria in infected patients^22^. Intravascular hemolysis produces methemoglobin products, with elevated levels in patients who either died or survived with neurological sequelae^23^. Understanding this association is crucial, as it could influence clinical outcomes and management strategies for malaria patients. Although some studies have explored the association between malaria and methemoglobin, findings have often been inconclusive or have varied across different geographical and demographic settings^19,20,22^. This systematic review and meta-analysis aimed to synthesize existing data to provide a more comprehensive and cohesive understanding of the association between malaria infections and severity-related alterations in methemoglobin levels. This study aims to shed light on potential diagnostic, prognostic, and therapeutic implications in malaria management.

## Methods

### Protocol and registration

The protocol of the systematic review and meta-analysis followed the Preferred Reporting Items for Systematic Reviews and Meta-Analyses guidelines^24^. The protocol was registered at PROSPERO (registration number: CRD42023468210)**.**

### Systematic review question

The systematic review questions were developed using the Population, Exposure, Comparator, Outcome (PECO) framework^25^. The primary question was, “In patients with malaria (P), how does the presence of *Plasmodium* infection (E), compared to those without the infection (C), affect the methemoglobin levels (O)?” The secondary question was, “How does the methemoglobin level vary in severe malaria compared to those with uncomplicated malaria?”.

### Search strategy

Several databases, including Ovid, ProQuest, Embase, Scopus, MEDLINE, and PubMed, were comprehensively searched to identify relevant studies that evaluated methemoglobin levels in patients with malaria. The general search strategy used for querying the databases was “Methemoglobin AND “(‘malaria’ OR ‘plasmodium’ OR ‘Plasmodium Infection’ OR ‘Remittent Fever’ OR ‘Marsh Fever’ OR ‘Paludism’).” The specific search strategies were used for individual databases (Table S1). In addition to the main databases, a search on Google Scholar were conducted to identify any additional records that were not indexed in the main databases. Only the first 200 articles from Google Scholar were screened to identify potentially relevant studies, as suggested previously^26^. Moreover, reference lists of included studies were reviewed to ensure that no relevant studies were missed. The searches were conducted from inception to September 29, 2023. The searches were not limited to publication year or the language of articles.

### Selection criteria

Studies were selected based on specific inclusion and exclusion criteria. Human studies that evaluated methemoglobin levels in patients with malaria and those that reported on methemoglobin levels either in patients with severe or uncomplicated malaria or provided a comparison group were included. In vitro studies, animal studies, and studies that lacked specific information on methemoglobin in malaria or only concerned methemoglobin levels after treatment were excluded. Reviews, assay developments, computational models, case reports or series, meta-analyses, conference abstracts, and studies with nonextractable data or that merely presented methemoglobin in malaria without a comparison group were also excluded.

### Study selection and data extraction

Duplicate records were removed before screening, and the remaining records were screened for eligibility based on the inclusion and exclusion criteria. Full-text articles of potentially relevant studies were retrieved and assessed for final inclusion. Data was extracted from the included studies regarding the publication year, study design, study location, *Plasmodium* species, age range of participants, clinical presentation of malaria, severity status, method for measuring methemoglobin, and *Plasmodium* detection. Furthermore, methemoglobin levels in different groups of patients with malaria were extracted. Study selection and data extraction were performed independently by two reviewers (KUK, MK); a third reviewer (AM) resolved disagreements.

### Quality assessment

A critical appraisal of studies included in this study was carried out using the Joanna Briggs Institute (JBI) checklist, which was specific for each study design. This ensured methodological quality of the selected studies in this review^27^. For cross-sectional studies, the JBI checklist emphasizes clear inclusion criteria, detailed descriptions of study subjects and settings, valid exposure and outcome measurements, and appropriate handling of confounding factors and statistical analysis. Cohort studies should have comparable groups, ensure participants are free from the outcome at the start, and implement reliable outcome measurements with suitable statistical handling. For case–control studies, the guidelines emphasize the importance of having comparable groups, consistent criteria for case and control identification, and accounting for confounding factors. Quasi-experimental studies should provide a distinct cause-and-effect relationship, have mechanisms to control confounders, ensure blinded outcome assessments, and employ appropriate statistical techniques. Each item or question on the checklist can be answered with “Yes,” “No,” “Unclear,” or “Not Applicable.” Two reviewers (KUK, MK) conducted the quality assessment independently; disagreements were resolved through discussion.

### Data syntheses and statistical analysis

Qualitative synthesis^28^ was performed to evaluate the difference in methemoglobin levels between patients with malaria and those without malaria. Furthermore, methemoglobin levels were compared between those with severe malaria and those with uncomplicated malaria. The aforementioned comparison was based on independent covariates, including publication year, study design, study area, *Plasmodium* species, age group, symptomatic status, severity status, and method of malaria detection. For the meta-analysis, the primary outcome of interest was the pooled standardized mean difference in methemoglobin levels between patients with malaria and those without malaria and between patients with severe malaria and those with uncomplicated malaria. Hedges’ g and its 95% confidence interval (CI) represented the pooled effect estimate. The heterogeneity among studies was quantified using the *I*^2^ statistic, wherein *I*^2^ values > 50% indicated significant heterogeneity^29^. In cases of significant heterogeneity, meta-regression analysis was conducted to identify potential sources of heterogeneity; furthermore, this analysis was conducted if at least 6 to 10 studies were selected for the meta-analysis^30^. Subgroup analyses were conducted based on predefined criteria, such as publication year, study design, study location, *Plasmodium* species, age group, symptomatic status, severity status, and method of *Plasmodium* identification. A sensitivity analysis was conducted using the leave-one-out method to evaluate the stability and reliability of the findings from a meta-analysis by assessing the influence of individual studies on the overall results^31^. All analyses were performed using the Stata v17.0 software (StataCorp, College Station, TX). A *P*-value < 0.05 was considered as statistically significant.

## Results

### Search results

**Figure 1:**
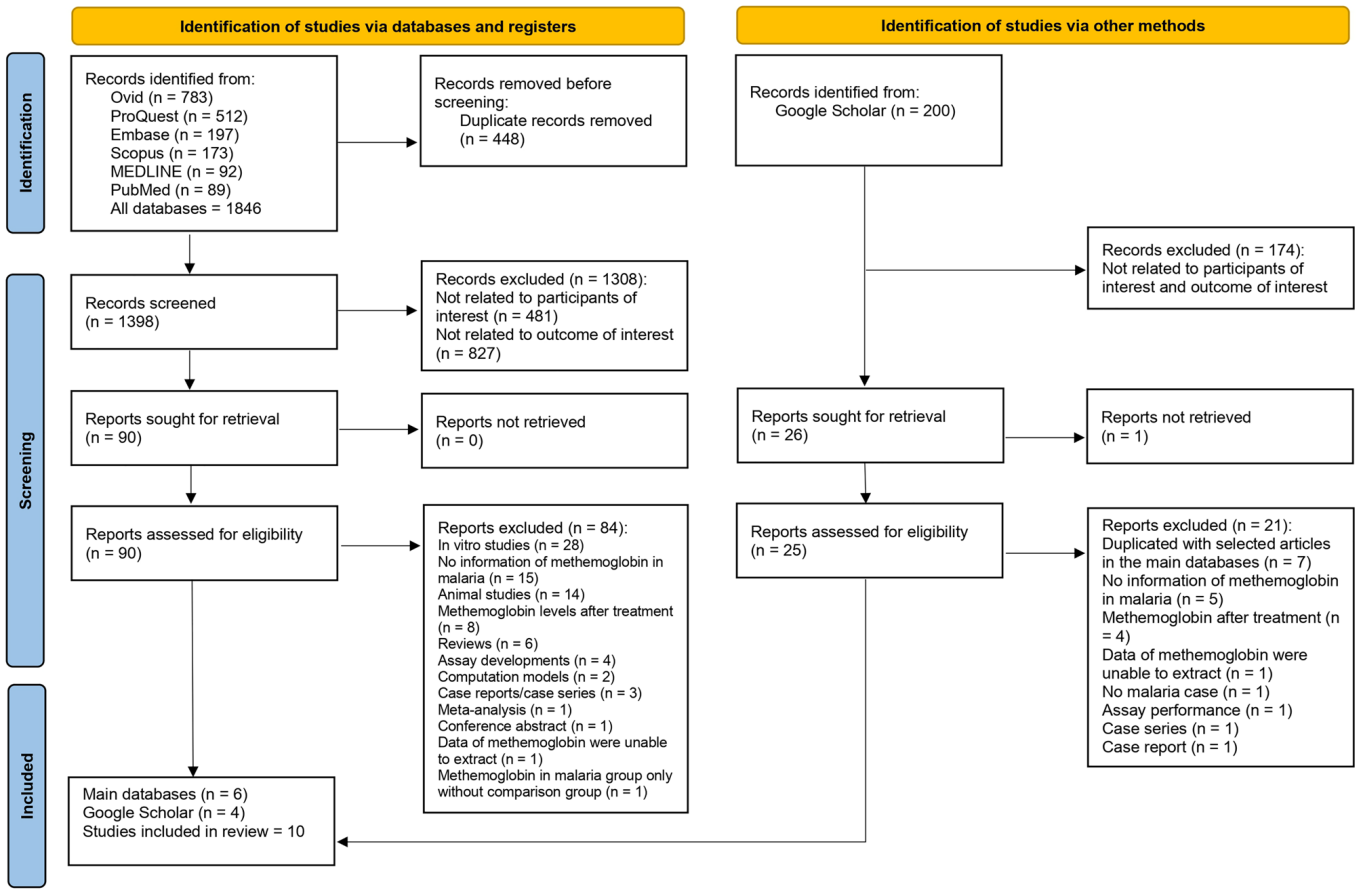
Study flow diagram.

Of the total 1846 studies identified from databases, including Ovid (n = 783), ProQuest (n = 512), Embase (n = 197), Scopus (n = 173), MEDLINE (n = 92), and PubMed (n = 89), 448 duplicate records were removed prior to screening, and 1398 records were screened. Of these, 1308 were excluded for being unrelated to the participants of interest (n = 481) or the outcome of interest (n = 827). The remaining 90 reports were assessed for eligibility. Of these 90 reports, 84 were excluded for being in vitro studies (n = 28), lacking information on methemoglobin in malaria (n = 15), being animal studies (n = 14), assessing methemoglobin levels after treatment (n = 8), and for several other specific reasons such as being reviews, assay developments, computational models, case reports or series, meta-analyses, conference abstracts, or having data that were not extractable or only featured methemoglobin in malaria without a comparison group., Google Scholar provided four studies in addition to the main databases. Eventually, 10 studies were included in this review^19,20,22,23,32–37^ (Fig. 1).

### Characteristics of included studies

Of the 10 studies selected for the review, 2 (20%) were published before 2000^23,33^, 1(10%) was published between 2000 and 2009^36^, 6 (60%) were published between 2010 and 2019^19,20,22,32,34,37^, and 1 (10%) was published between 2020 and 2023^35^. Regarding study designs, 2 (20%) were cross-sectional studies^23,37^, 4 (40%) were case–control studies^22,33,34,36^, 3 (30%) were cohort studies^19,20,35^, and 1 (10%) was a quasi-experimental study^32^. Four studies (40%) were conducted in Asia^19,22,33,35^, with 2 (20%) in India^19,35^, 1 (10%) in Indonesia^22^, and 1 (10%) in Turkey^33^. Six (60%) originated from Africa^20,23,32,34,36,37^, including 4 (40%) from Nigeria^32,34,36,37^, 1 (10%) from Gabon^20^, and 1(10%) from Tanzania^23^. *P. falciparum* and *P. vivax* were the primary *Plasmodium* species investigated. Specifically, 6 studies (60%) focused on *P. falciparum*^20,22,23,35–37^ and 1 study (10%) focused on *P. vivax*^33^; the remaining 3 studies (30%) did not specify the *Plasmodium* species^19,32,34^. Three studies (30%) involved children^20,23,36^, 5 (50%) involved adults^22,32,33,35,37^, and 2 (20%) did not specify the age group^19,34^. Eight (80%) studies, a majority, enrolled patients with symptomatic malaria^19,20,22,23,33–36^, 1 (10%) enrolled a patient with asymptomatic malaria^37^, and 1 (10%) did not specify the symptomatic status of the patient. Two studies (20%) enrolled patients with severe malaria^33,36^; 4 (40%) enrolled patients with both severe and uncomplicated malaria^19,20,34,35^; 1 (10%) was focused on severe and moderately severe malaria^22^; 1 (10%) enrolled patients with severe, uncomplicated, and asymptomatic malaria^23^; 1 (10%) enrolled patients with asymptomatic malaria alone^37^; and 1 (10%) did not specify the severity status of patients^32^. Six studies (60%) used the microscopic method for malaria detection^20,23,33,34,36,37^; 2 (20%) used a combination of the microscopic method, quantitative buffy coat, and the rapid diagnostic test (RDT)^19,35^; 1 (10%) combined the microscopic method with RDT^22^, and 1 (10%) did not specify the method of malaria detection^32^ (Table 1, Table S2).

**Table 1:** Summary characteristics of the included studies.

Characteristics	n (10 studies)	**%**	References
Publication year			
Before 2000	2	20	^23,33^
2000–2009	1	10	^36^
2010–2019	6	60	^19,20,22,32,34,37^
2020–2023	1	10	^35^
Study designs			
Cross-sectional	2	20	^23,37^
Case–control	4	40	^22,33,34,36^
Cohort	3	30	^19,20,35^
Quasi-experimental	1	10	^32^
Study areas			
Asia	4	40	^19,22,33,35^
India	2	20	^19,35^
Indonesia	1	10	^22^
Turkey	1	10	^33^
Africa	6	60	^20,23,32,34,36,37^
Nigeria	4	40	^32,34,36,37^
Gabon	1	10	^20^
Tanzania	1	10	^23^
*Plasmodium* species			
*P. falciparum*	6	60	^20,22,23,35–37^
*P. vivax*	1	10	^33^
Not specified	3	30	^19,32,34^
Participants			
Children	3	30	^20,23,36^
Adults	5	50	^22,32,33,35,37^
Not specified	2	20	^19,34^
Symptomatic status			
Symptomatic malaria	8	80	^19,20,22,23,33–36^
Asymptomatic malaria	1	10	^37^
Not specified	1	10	^32^
Severity status			
Severe malaria	2	20	^33,36^
Severe and uncomplicated malaria	4	40	^19,20,34,35^
Severe and moderately severe malaria	1	10	^22^
Severe, uncomplicated, asymptomatic malaria	1	10	^23^
Asymptomatic malaria	1	10	^37^
Not specified	1	10	^32^
Methods for malaria detection			
Microscopic method	6	60	^20,23,33,34,36,37^
Microscopic method, quantitative buffy coat, RDT	2	20	^19,35^
Microscopic method, RDT	1	10	^22^
Not specified	1	10	^32^

*RDT* rapid diagnostic test.

### Quality of included studies

For analytical cross-sectional studies, one met all criteria^23^, while another had uncertainties about confounding factors^37^. In case–control studies, 3^33,34,36^ had issues regarding confounding factors, while one met all criteria^22^. In cohort studies, 3 had unclear follow-up and confounding factor issues^19,20,35^. The quasi-experimental study was clear on cause–effect relationships and had consistent outcome measurements but unclear follow-up completeness^32^ (Table S3).

### Thematic synthesis for methemoglobin levels in malaria

Methemoglobin levels in different groups of patients with malaria are shown in Table 2. Based on geographical overview, studies in Africa, such as in Tanzania^23^, Nigeria^32,34,36,37^, and Gabon^20^, consistently showed an increase in methemoglobin levels in malaria patients. Similarly, studies in Asia, such as in India^19,35^, Turkey^33^, and Indonesia^22^, also indicated elevated methemoglobin levels in those with malaria. Based on *Plasmodium* species, Anstey et al. reported elevated methemoglobin levels in cases of severe, uncomplicated, and cerebral malaria caused by *P. falciparum* in Tanzanian children^23^. In Gabon, Hänscheid et al. reported increased methemoglobin levels in children suffering from severe and uncomplicated *P. falciparum* malaria^20^. Yeo et al. from Indonesia found increased methemoglobin levels proportional to malaria disease severity caused by *P. falciparum* in adults^22^. They also observed no direct correlation between methemoglobin levels and the degree of parasitemia. In Nigeria, Zama et al. and Uko et al. reported that *P. falciparum* infections in adults and children, respectively, were associated with significantly higher methemoglobin levels compared to uninfected controls^36,37^. Both studies also found a direct correlation between methemoglobin levels and the degree of parasitemia. A study from Turkey by Erel et al. focusing on adult cases of severe malaria caused by *P. vivax* revealed that methemoglobin levels were significantly elevated in patients compared to uninfected controls^33^.

**Table 2:** Comparison of methemoglobin levels in different groups of patients with malaria.

Authors	Study location	Continent	*Plasmodium* spp.	Age range (years)	Clinical malaria (severe, uncomplicated, mild)	Clinical malaria (symptomatic or asymptomatic)	Methemoglobin levels
Anstey et al., 1996^23^	Tanzania	Africa	*P. falciparum*	6 months–9 years	Severe, uncomplicated, asymptomatic	Symptomatic and asymptomatic	Methemoglobin levels were significantly higher in patients with malaria (severe malaria, uncomplicated malaria, cerebral malaria) than uninfected controls
Behera et al., 2016^19^	India	Asia	Not specified	Not specified	Severe, uncomplicated	Symptomatic	1. Methemoglobin levels were significantly higher in severe malaria than in uncomplicated malaria. 2. Methemoglobin levels were significantly higher in patients with malaria than in uninfected controls. 3. A direct correlation was observed between methemoglobin levels and the degree of parasitemia
Chikezie PC, 2018^32^	Nigeria	Africa	Not specified	Malaria (45): 21–34, nonmalaria (43): 20–28 years	Not specified	Not specified	Methemoglobin levels were significantly higher in patients with malaria than in uninfected controls
Erel et al., 1997^33^	Turkey	Asia	*P. vivax*	15–35 years	Severe	Symptomatic malaria	Methemoglobin levels were significantly higher in patients with malaria than in uninfected controls
Hänscheid et al., 2014^20^	Gabon	Africa	*P. falciparum*	Not specified	Severe, uncomplicated	Symptomatic malaria	Methemoglobin levels were significantly higher in patients with malaria than in uninfected controls
Ifeanyi et al., 2013^34^	Nigeria	Africa	Not specified	Not specified	Severe, uncomplicated	Symptomatic malaria	1. Methemoglobin levels were significantly higher in severe malaria than in uncomplicated malaria. 2. Methemoglobin levels were significantly higher in patients with malaria than in uninfected controls
Karua et al., 2020^35^	India	Asia	*P. falciparum*	20–50 years	Severe, uncomplicated	Symptomatic malaria	1. Methemoglobin levels were significantly higher in severe malaria than in uncomplicated malaria. 2. Methemoglobin levels were significantly higher in patients with malaria than in uninfected controls. 3. A direct correlation was observed between methemoglobin levels and the degree of parasitemia
Uko et al., 2003^36^	Nigeria	Africa	*P. falciparum*	11 months–15 years	Severe	Symptomatic malaria	1. Methemoglobin levels were significantly higher in patients with malaria than in uninfected controls. 2. A direct correlation was observed between methemoglobin levels and the degree of parasitemia
Yeo et al., 2013^22^	Indonesia	Asia	*P. falciparum*	≥ 18 years	Severe and moderately severe	Symptomatic malaria	1. Methemoglobin levels were also increased in proportion to malaria disease severity. 2. No association between methemoglobin levels and degree of parasitemia
Zama et al., 2013^37^	Nigeria	Africa	*P. falciparum*	18–45 years	Asymptomatic	Asymptomatic malaria	1. Methemoglobin levels were significantly higher in patients with malaria than in uninfected controls. 2. A direct correlation was observed between methemoglobin levels and the degree of parasitemia

Based on clinical presentation, higher methemoglobin levels were noted in those with symptomatic and asymptomatic malaria than in uninfected controls, as observed in 2 studies^23,37^. Furthermore, several studies^20,23,34,35^ revealed that methemoglobin levels were significantly elevated in severe malaria cases compared to uncomplicated malaria cases. Based on age group dynamics, studies that enrolled children^20,23,36^ and adults^22,32,33^ found consistently elevated methemoglobin levels in malaria cases. For parasitemia correlation, 3 studies^19,36,37^ observed a direct correlation between methemoglobin levels and the degree of parasitemia. However, a study by Yeo et al. from Indonesia found no such association^22^.

### Methemoglobin levels between patients with malaria and without malaria

**Figure 2:**
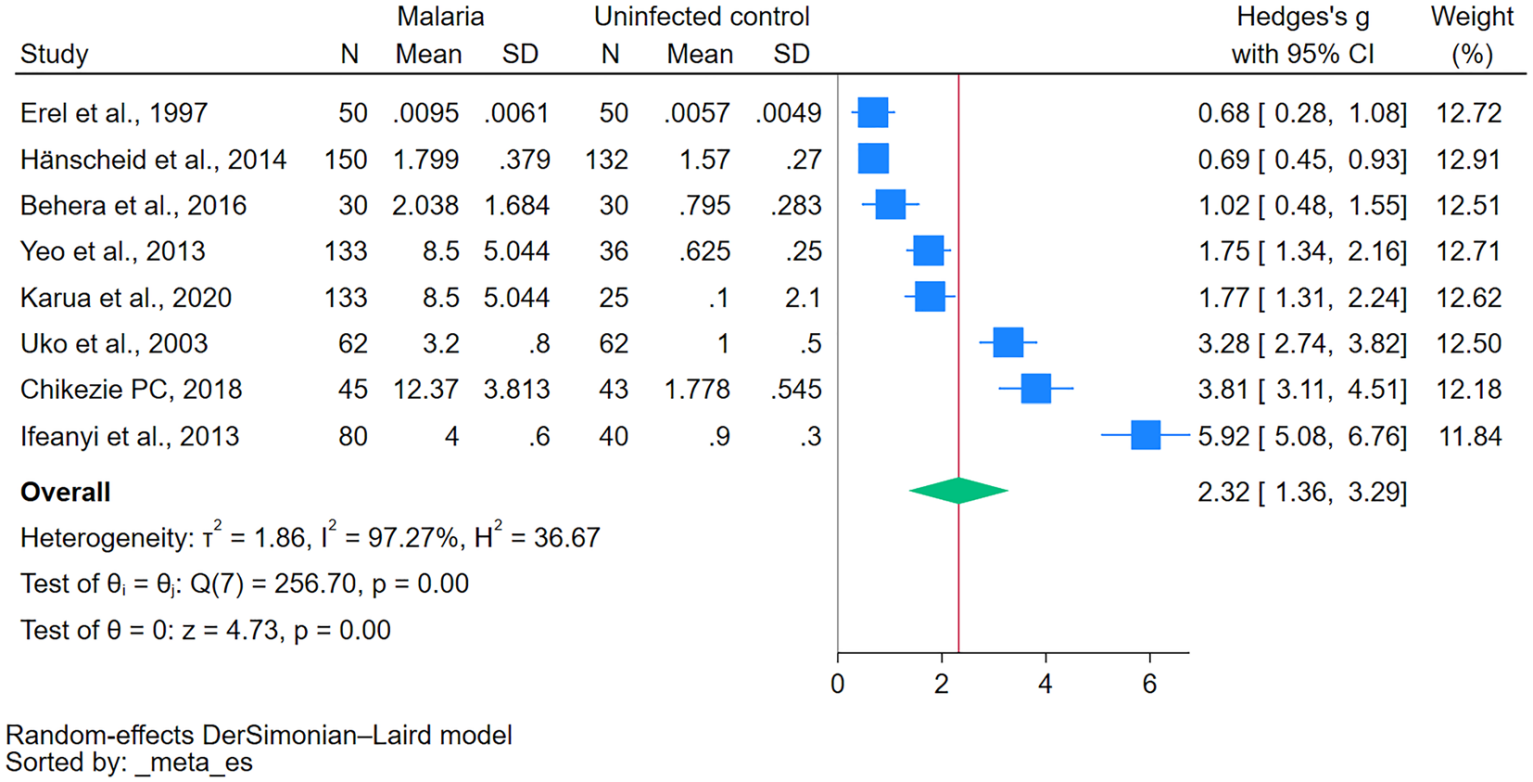
The difference in methemoglobin levels between patients with and without malaria. *Blue box* effect estimate, *green diamond* pooled effect estimate, *N* number of participants, *Mean* mean methemoglobin level, *SD* standard deviation, *CI* confidence interval.

The difference in methemoglobin levels between patients with malaria and those without malaria was estimated using the data from 8 studies^19,20,22,32–36^. The results demonstrated increased methemoglobin levels in patients with malaria compared to those without malaria (*P* < 0.001, Hedges’ g 2.32, 95% CI 1.36–3.29, *I*^2^ 97.27, 8 studies, Fig. 2). Because the results were heterogeneous, meta-regression analysis was conducted to identify factors that affected the pooled effect estimate. The results revealed that only the country significantly affected the pooled effect estimate (*P* < 0.001, R^2^ = 51.47, Table S4).

Subgroup analyses evaluated the difference in methemoglobin levels between patients with and without malaria. Notably, studies published between 2010 and 2019 showed a significant increase in methemoglobin levels in patients with malaria (*P* < 0.01)^19,20,22,32^. Regarding study design, the case–control and cohort studies revealed a significant rise in methemoglobin levels for patients with malaria (*P* < 0.01)^19,20,22,33–36^. Geographically, studies from Asia and Africa demonstrated significantly elevated methemoglobin levels in patients with malaria (*P* < 0.01)^19,20,22,32–36^. When analyzed by age group, a significant increase in methemoglobin levels was observed among studies that enrolled adults with malaria (*P* < 0.01)^22,32,33,35^ but not in those that enrolled children (*P* = 0.13)^20,36^.

Subgroup analysis based on the *Plasmodium* species revealed significantly elevated methemoglobin levels in patients with *P. falciparum* malaria compared to those without infection (*P* < 0.01). Furthermore, in terms of clinical presentation, patients with symptomatic malaria demonstrated significantly higher methemoglobin levels than the uninfected controls (*P* < 0.01). When considering methods of *Plasmodium* identification, using the microscopic method, either alone or combined with quantitative buffy coat and RDT, showed a significant rise in methemoglobin levels among malaria patients (*P* < 0.01) (Table 3).

**Table 3:** Subgroup analyses of the difference in methemoglobin levels between patients with malaria and those without malaria (uninfected controls).

Subgroup analyses	*P*-value	Hedges’ g (95% CI)	*I*^2^ (%)	Number of studies	References
Publication years					
2020–2023	N/A	1.77 (1.31–2.24)	N/A	1	^35^
2010–2019	< 0.01	2.60 (1.13–4.06)	97.96	4	^19,20,22,32^
2000–2009	N/A	3.28 (2.74–3.82)	N/A	1	^36^
Before 2000	N/A	1.24 (0.28–1.08)	N/A	1	^33^
Study design					
Case–control study	< 0.01	2.87 (1.10–4.65)	97.98	4	^22,33,34,36^
Cohort study	< 0.01	1.14 (0.47–1.81)	87.90	3	^19,20,35^
Quasi-experimental study	N/A	3.81 (3.11–4.51)	N/A	1	^32^
Continent					
Africa	< 0.01	3.40 (1.11–5.70)	98.71	4	^20,32,34,36^
Asia	< 0.01	1.31 (0.74–1.87)	84.01	4	^19,22,33,35^
Age group					
Children	0.13	1.97 (–0.57–4.51)	98.66	2	^20,36^
Adults	< 0.01	1.97 (0.92–3.03)	94.97	4	^22,32,33,35^
Not specified	0.16	3.46 (–1.35–8.26)	98.93	2	^19,34^
*Plasmodium* species					
*P. falciparum*	< 0.01	1.85 (0.80–2.90)	96.49	4	^20,22,34,35^
*P. vivax*	N/A	0.68 (0.28–1.08)	N/A	1	^33^
Not specified	0.01	3.57 (0.73–6.41)	98.09	3	^19,32,34^
Clinical presentation					
Symptomatic malaria	< 0.01	2.11 (1.14–3.08)	97.17	7	^19,20,22,33–36^
Not specified	N/A	3.81 (3.11–4.51)	N/A	1	^32^
Methods for *Plasmodium* identification					
Microscopic method	< 0.01	2.60 (0.82–4.39)	98.52	4	^20,33,34,36^
Microscopic method, RDT	N/A	1.75 (1.34–2.16)	N/A	1	^22^
Microscopic method, Quantitative buffy coat, RDT	< 0.01	1.41 (0.66–2.15)	77.27	2	^19,35^
Not specified	N/A	3.81 (3.11–4.51)	N/A	1	^32^

*CI* confidence interval, *N/A* not assessed, *RDT* rapid diagnostic test.

### Methemoglobin levels between patients with severe malaria and uncomplicated malaria

**Figure 3:**
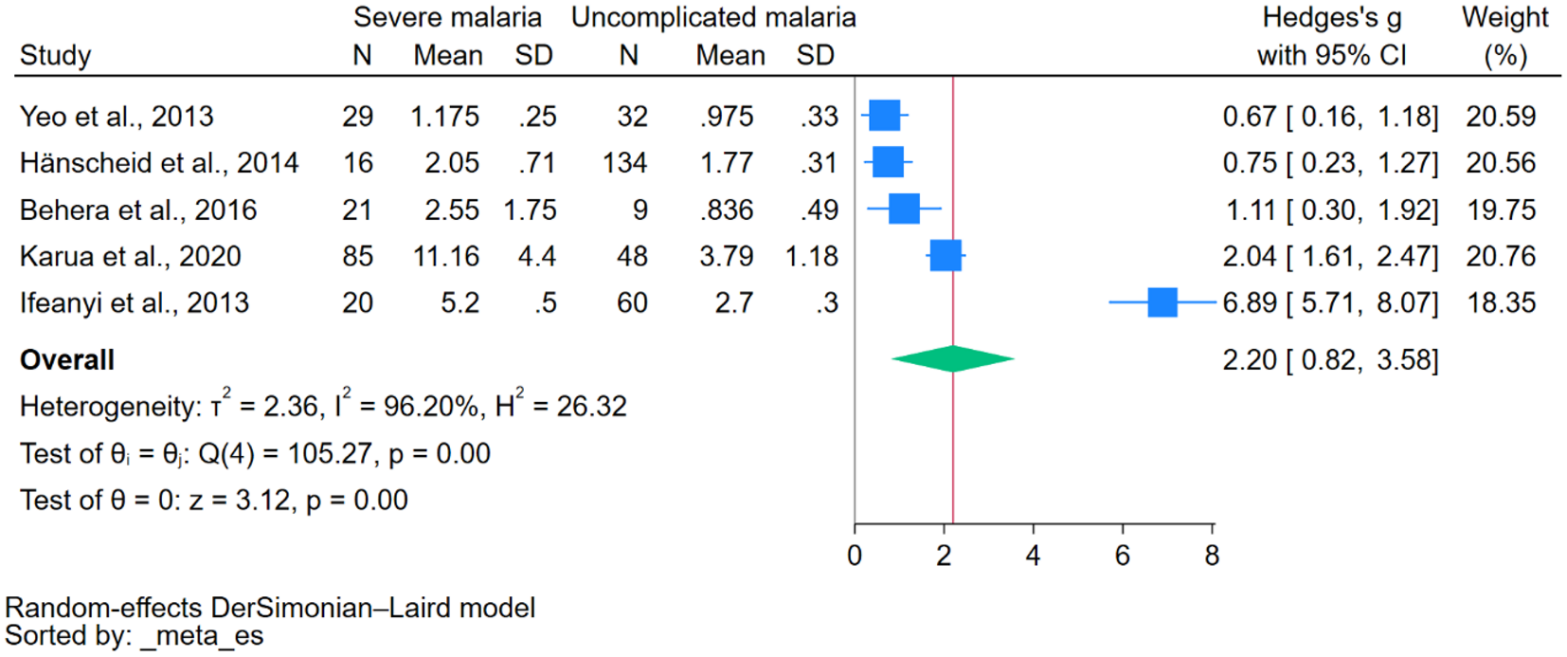
The difference in methemoglobin levels between patients with severe malaria and those with uncomplicated malaria. *Blue box* effect estimate, *green diamond* pooled effect estimate, *N* number of participants, *Mean* mean methemoglobin level, *SD* standard deviation, *CI* confidence interval.

The difference in methemoglobin levels between patients with severe malaria and those with uncomplicated malaria was evaluated using data from 5 studies^19,20,22,34,35^. The results revealed elevated methemoglobin levels in patients with severe malaria compared to those with uncomplicated malaria (*P* < 0.001, Hedges’ g 2.20, 95% CI 0.82–3.58, *I*^2^ 96.20, 5 studies, Fig. 3). Meta-regression and subgroup analyses were not performed because of the limited number of studies in the meta-analysis.

### Sensitivity analysis

**Figure 4:**
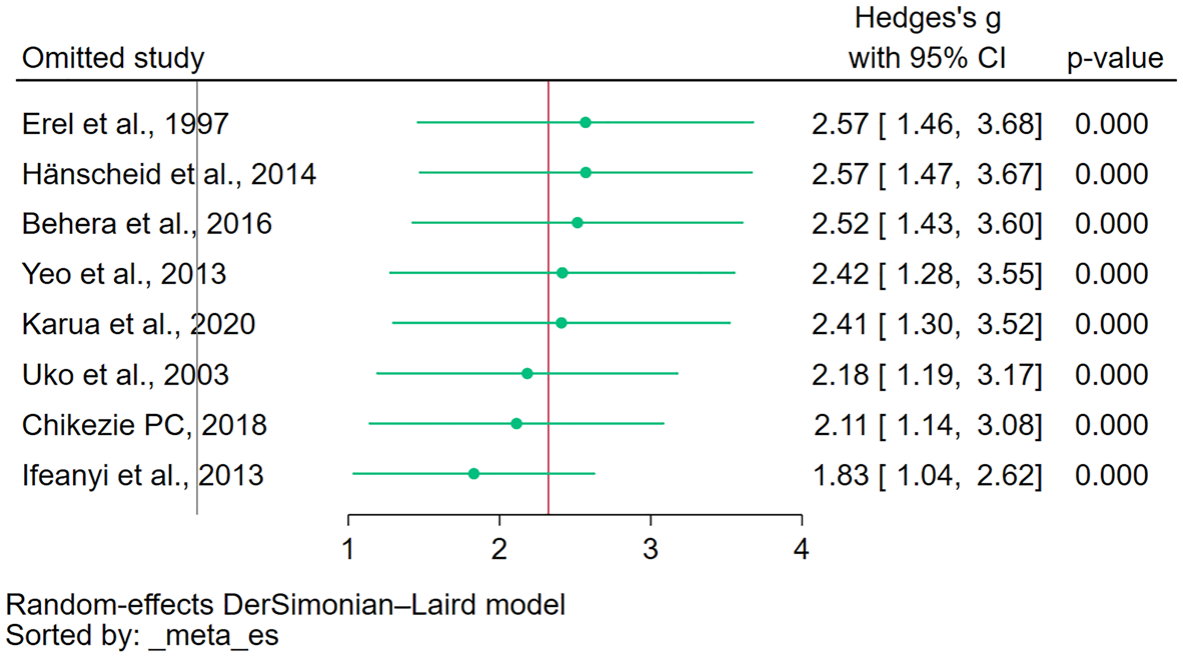
The leave-one-out meta-analysis revealed the robustness of the meta-analysis results that showed significantly increased methemoglobin levels in patients with malaria compared to those without malaria. It confirmed that these significant findings remained consistent even when individual studies were excluded one by one from the analysis. *Green dot* pooled effect estimate, *green line* confidence interval, *CI* confidence interval.

**Figure 5:**
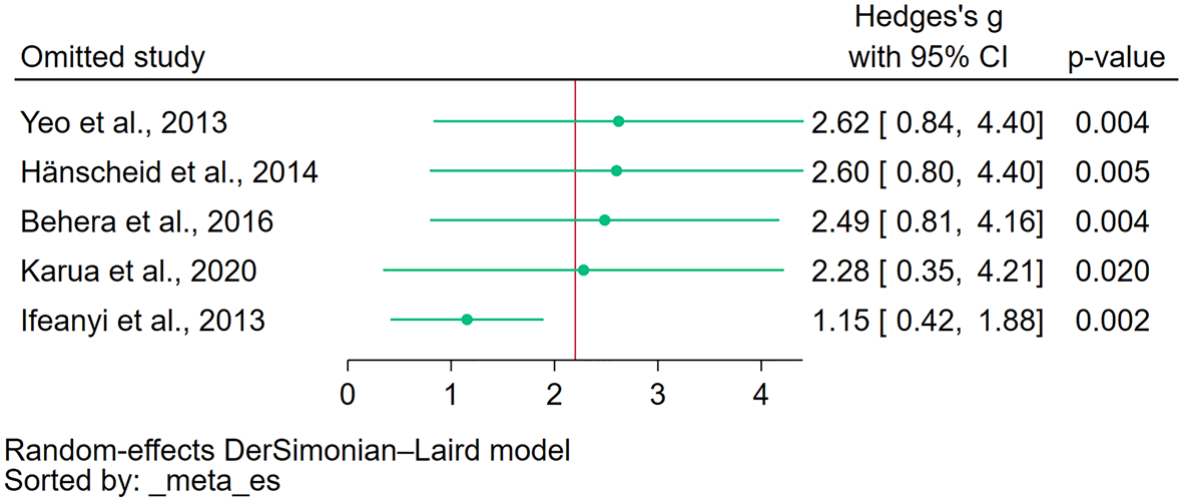
The leave-one-out meta-analysis revealed the robustness of the meta-analysis results that showed significantly increased methemoglobin levels in patients with severe malaria compared to those with uncomplicated malaria. It confirmed that these significant findings remained consistent even when individual studies were excluded one by one from the analysis. *Green dot* pooled effect estimate, *green line* confidence interval, *CI* confidence interval.

The leave-one-out meta-analysis revealed the robustness of the meta-analysis results that showed significantly increased methemoglobin levels in patients with malaria compared to those without malaria (Fig. 4) and significantly increased methemoglobin levels in patients with severe malaria compared to those with uncomplicated malaria (Fig. 5). The leave-one-out method confirmed that these significant findings remained consistent even when individual studies were excluded one by one from the analysis.

## Discussion

The systematic review primarily examined the methemoglobin levels in patients with malaria. The comprehensive search across multiple databases yielded 10 studies that met the eligibility criteria^19,20,22,23,32–37^. The review results can be used to draw several key observations and insights. First, the geographical spread of the studies reflects the endemic regions where malaria is prevalent. Asia and Africa, regions significantly burdened by malaria, have been actively involved in research to understand the nuances of this disease, as evidenced by the studies included in the review. Most studies were published between 2010 and 2019^19,20,22,32,34,37^, underscoring the continued research interest in this period. Regarding study designs, case–control studies made up the largest proportion. This approach is advantageous for understanding associations between disease states and potential risk factors, in this case, the methemoglobin levels.

A significant observation from qualitative synthesis was the consistent rise in methemoglobin levels in patients with malaria across diverse geographical locations, such as Tanzania, Nigeria, India, Turkey, and Indonesia. This uniformity suggests a strong biological link between malaria infection and elevated methemoglobin levels. Furthermore, when the focus was narrowed down to specific *Plasmodium* species, such as *P. falciparum*, the observation remained consistent, with increased methemoglobin levels reported in most of the studies. Clinically, according to the data from the included studies, there is insufficient evidence to determine whether higher methemoglobin levels are present in both symptomatic and asymptomatic malaria compared to absent infection. Importantly, the severity of malaria also seems to influence methemoglobin levels, with those having severe malaria showing consistently higher levels compared to those with uncomplicated malaria. Furthermore, increased methemoglobin levels have been associated with increased mortality^19^. Elevated methemoglobin levels can reduce the blood’s oxygen-carrying capacity, leading to hypoxia and vice versa^19^. The subgroup analysis revealed a significant age-related variation in methemoglobin levels among patients with malaria. Specifically, studies focusing on adults with malaria showed a marked increase in methemoglobin levels, unlike those in children, where this increase was not statistically significant. This difference could be attributed to immunological variances between adults and children or to diverse levels of exposure or responses to the *Plasmodium* parasite^38,39^. Furthermore, it is plausible that differences in the metabolic pathways responsible for methemoglobin production and clearance exist between these age groups. This can be attributed to the fact that methemoglobin levels are typically higher in children than in adults, which may be because of the lower amounts of soluble cofactor cytochrome b5 and reduced activity of the cytochrome b5 reductase enzyme in their red blood cells, increasing their risk of methemoglobinemia^40^.

The results concerning the association of methemoglobin levels and parasitemia were mixed. Although some studies found a direct association^19,36,37^, one study from Indonesia did not observe such an association^22^. Such disparities may arise from various factors, including differences in study design, sample size, and population characteristics. Regarding study quality, while many met most of the criteria, certain aspects, such as confounding factors and follow-up completeness, remained areas of concern in some studies. This highlights the need for a more rigorous approach in future research endeavors.

The meta-analysis conclusively demonstrated a significant elevation in methemoglobin levels in patients with malaria compared to those without malaria. This observation was consistent across the reviewed studies. Sensitivity analysis further confirmed its reliability. In vitro studies have shed light on possible mechanisms: methemoglobin-driven red blood cell aggregation, coupled with the generation of reactive oxygen species (ROS) in the external microenvironment, seems pivotal in the pathophysiological effects observed during malaria^41^. Moreover, methemoglobin-treated endothelial cells exhibited heightened ROS levels, suggesting that methemoglobin can boost the cytoadherence of uninfected red blood cells to these cells^42^. This early ROS increase driven by methemoglobin contributes to the osmotic fragility and subsequent destruction of red blood cells^43^. Methemoglobin has been reported to mediate toxicity toward macrophages, which might lead to a weakened immune response during malaria^44^. In patients with malaria, increased methemoglobin levels have been observed after treatment with antimalarial drugs like primaquine^45–47^. However, in patients with normal glucose-6-phosphate dehydrogenase (G-6-PD) activity and in nonpregnant women, high doses of primaquine were not associated with elevated methemoglobin levels^45,46^. A decline in methemoglobin levels after treatment following the clearance of parasites was due to the partial restoration of the redox balance inside red blood cells and the consequent reduction of the methemoglobin level^45^.

The study has some limitations. First, the observed heterogeneity in the results could be attributed to variations in study designs, methodologies, and population demographics. Second, the limited number of studies available for certain subgroup analyses, such as the difference in methemoglobin levels between patients with severe and uncomplicated malaria, may have affected the conclusiveness of the results. Third, because of the limited number of studies, publication bias could not be assessed, potentially influencing the conclusions drawn from the meta-analysis. The systematic review accentuated the global significance of understanding the association between malaria and methemoglobin levels, especially in malaria-endemic regions. Elevated methemoglobin levels in patients with malaria, regardless of their symptomatic status, emerge as a potential biomarker, which, when correlated with severe malaria and increased mortality, underscored its clinical importance. Insights into the effects of antimalarial drugs, notably primaquine, suggested the necessity of vigilant drug administration. Furthermore, the observed pathophysiological effects highlighted possible therapeutic intervention areas. Despite the clear associations, discrepancies in some findings and inherent study limitations indicate the need for more rigorous, expansive research to solidify these observations and influence health policies in affected regions.

## Conclusion

In conclusion, this systematic review and meta-analysis revealed increased methemoglobin levels in patients with *P. falciparum* and *P. vivax* infections, with a notable association between elevated methemoglobin levels and severe malaria. Future research should focus on elucidating the specific mechanisms by which changes in methemoglobin levels are related to infections by *P. falciparum* and *P. vivax*, particularly in terms of severity, and how these alterations could potentially impact patient management and treatment outcomes.

### Supplementary Information

Supplementary Table S1.

Supplementary Table S2.

Supplementary Table S3.

Supplementary Table S4.

## Data Availability

All data relating to the present study are available in this manuscript and supplementary files.
