# Supplementary material for: Methemoglobin levels in malaria: a systematic review and meta-analysis of its association with Plasmodium falciparum and Plasmodium vivax infections and disease severity
Source: Sci Rep. 2024 Feb 8;14:3276. doi: 10.1038/s41598-024-53741-6 (PMC10853561; doi:10.1038/s41598-024-53741-6)
Supplement: Supplementary file 1 — Supplementary Table S1. [file 41598_2024_53741_MOESM1_ESM.docx]

**Methemoglobin levels in malaria: a systematic review and meta-analysis of its association with *Plasmodium falciparum* and *Plasmodium vivax* infections and disease severity**

Manas Kotepui^1^*, Aongart Mahittikorn^2*^, Polrat Wilairatana^3^, Frederick Ramirez Masangkay^4^, Kinley Wangdi^5^, Kwuntida Uthaisar Kotepui^1^

^1^Medical Technology, School of Allied Health Sciences, Walailak University, Thasala, Nakhon Si Thammarat 80160, Thailand

^2^Department of Protozoology, Faculty of Tropical Medicine, Mahidol University, Bangkok 10400, Thailand

^3^Department of Clinical Tropical Medicine, Faculty of Tropical Medicine, Mahidol University, Bangkok 10400, Thailand

^4^Department of Medical Technology, Faculty of Pharmacy, University of Santo Tomas, Manila 1000, Philippines

^5^QIMR Medical Research Institute, 300 Herston Road, Herston QLD 4006 Australia

*Corresponding author

Manas Kotepui manas.ko@wu.ac.th

Aongart Mahittikorn: aongart.mah@mahidol.ac.th

Frederick Ramirez Masangkay: frederick_masangkay2002@yahoo.com

Kinley Wangdi: kinley.wangdi@qimrberghofer.edu.au

Polrat Wilairatana: polrat.wil@mahidol.ac.th

Kwuntida Uthaisar Kotepui: kwuntida.ut@wu.ac.th

**General keywords**

Methemoglobin AND (malaria OR plasmodium OR “Plasmodium Infection“ OR “Remittent Fever“ OR “Marsh Fever“ OR Paludism)

PubMed 29 September 2023

| No. | Key concept | Search terms | Results |
| --- | --- | --- | --- |
| 1. | Methemoglobin | methemoglobin[Text Word] OR methemoglobin [MeSH Terms] | 6,226 |
| 2. | Malaria | malaria[Text Word] OR malaria[MeSH Terms] OR plasmodium[Text Word] OR “Plasmodium Infection“[Text Word] OR “Remittent Fever“[Text Word] OR “Marsh Fever“[Text Word] OR Paludism[Text Word] | 119,594 |
| 3. | 1 AND 2 | (methemoglobin[Text Word] OR methemoglobin [MeSH Terms]) AND (malaria[Text Word] OR malaria[MeSH Terms] OR plasmodium[Text Word] OR “Plasmodium Infection“[Text Word] OR “Remittent Fever“[Text Word] OR “Marsh Fever“[Text Word] OR Paludism[Text Word]) | 89 |

Embase 29 September 2023

| No. | Key concept | Search terms | Results |
| --- | --- | --- | --- |
| 1. | Methemoglobin | methemoglobin:ti,ab,kw,de OR methemoglobin/exp | 8,790 |
| 2. | Malaria | malaria:ti,ab,kw,de OR plasmodium:ti,ab,kw,de OR ‘Remittent Fever’:ti,ab,kw,de OR ‘Marsh Fever’:ti,ab,kw,de OR Paludism:ti,ab,kw,de OR malaria/exp | 158,029 |
| 3. | 1 AND 2 | (methemoglobin:ti,ab,kw,de OR methemoglobin/exp) AND (malaria:ti,ab,kw,de OR plasmodium:ti,ab,kw,de OR ‘Remittent Fever’:ti,ab,kw,de OR ‘Marsh Fever’:ti,ab,kw,de OR Paludism:ti,ab,kw,de OR malaria/exp) | 197 |

Scopus 29 September 2023

| No. | Key concept | Search terms | Results |
| --- | --- | --- | --- |
| 1. | Methemoglobin | TITLE-ABS-KEY (methemoglobin) | 9,797 |
| 2. | Malaria | TITLE-ABS-KEY ( malaria OR plasmodium OR "plasmodium infection" OR "remittent fever" OR "marsh fever" OR paludism ) | 158,891 |
| 3. | 1 AND 2 | ( TITLE-ABS-KEY (methemoglobin) ) AND ( TITLE-ABS-KEY ( malaria OR plasmodium OR "plasmodium infection" OR "remittent fever" OR "marsh fever" OR paludism ) ) | 173 |

Ovid 29 September 2023

| No. | Key concept | Search terms | Results |
| --- | --- | --- | --- |
| 1. | Methemoglobin AND Malaria | Methemoglobin AND (malaria OR plasmodium OR “Plasmodium Infection“ OR “Remittent Fever“ OR “Marsh Fever“ OR Paludism)  Filter: limit to ovid full text available and articles with abstracts and original articles | 783 |

MEDLINE 29 September 2023

| No. | Key concept | Search terms | Results |
| --- | --- | --- | --- |
| 1. | Methemoglobin AND Malaria | Methemoglobin AND (malaria OR plasmodium OR “Plasmodium Infection“ OR “Remittent Fever“ OR “Marsh Fever“ OR Paludism) | 92 |

ProQuest 29 September 2023

| No. | Key concept | Search terms | Results |
| --- | --- | --- | --- |
| 1. | Methemoglobin AND Malaria | Methemoglobin AND (malaria OR plasmodium OR “Plasmodium Infection“ OR “Remittent Fever“ OR “Marsh Fever“ OR Paludism) | 512 |
