# Supplementary material for: Methemoglobin levels in malaria: a systematic review and meta-analysis of its association with Plasmodium falciparum and Plasmodium vivax infections and disease severity
Source: Sci Rep. 2024 Feb 8;14:3276. doi: 10.1038/s41598-024-53741-6 (PMC10853561; doi:10.1038/s41598-024-53741-6)
Supplement: Supplementary file 4 — Supplementary Table S4. [file 41598_2024_53741_MOESM4_ESM.docx]

**Methemoglobin levels in malaria: a systematic review and meta-analysis of its association with *Plasmodium falciparum* and *Plasmodium vivax* infections and disease severity**

Manas Kotepui^1^*, Aongart Mahittikorn^2*^, Polrat Wilairatana^3^, Frederick Ramirez Masangkay^4^, Kinley Wangdi^5^, Kwuntida Uthaisar Kotepui^1^

^1^Medical Technology, School of Allied Health Sciences, Walailak University, Thasala, Nakhon Si Thammarat 80160, Thailand

^2^Department of Protozoology, Faculty of Tropical Medicine, Mahidol University, Bangkok 10400, Thailand

^3^Department of Clinical Tropical Medicine, Faculty of Tropical Medicine, Mahidol University, Bangkok 10400, Thailand

^4^Department of Medical Technology, Faculty of Pharmacy, University of Santo Tomas, Manila 1000, Philippines

^5^QIMR Medical Research Institute, 300 Herston Road, Herston QLD 4006 Australia

*Corresponding author

Manas Kotepui manas.ko@wu.ac.th

Aongart Mahittikorn: aongart.mah@mahidol.ac.th

Frederick Ramirez Masangkay: frederick_masangkay2002@yahoo.com

Kinley Wangdi: kinley.wangdi@qimrberghofer.edu.au

Polrat Wilairatana: polrat.wil@mahidol.ac.th

Kwuntida Uthaisar Kotepui: kwuntida.ut@wu.ac.th

**Table S4. Meta-regression analysis of covariates on the difference in methemoglobin levels between patients with malaria and uninfected controls**

| **Covariates** | ***P* value** | **tau^2^** | ***I^2^* (%)** | **R-squared (%)** | **Number of studies** |
| --- | --- | --- | --- | --- | --- |
| Publication years | 0.669 | 2.694 | 97.96 | 0.00 | 8 |
| Study design | 0.128 | 1.724 | 96.97 | 7.24 | 8 |
| Continent | 0.065 | 2.455 | 97.62 | 0.00 | 8 |
| Country | 0.0007 | 0.9021 | 90.55 | 51.47 | 8 |
| Age group | 0.579 | 2.724 | 97.81 | 0.00 | 8 |
| *Plasmodium* species | 0.150 | 2.019 | 97.37 | 0.00 | 8 |
| Clinical presentation | 0.231 | 1.645 | 97.17 | 11.50 | 8 |
| Diagnostic method for malaria | 0.644 | 2.639 | 98.07 | 0.00 | 8 |
